# Ravulizumab is Effective and Safe for Neuromyelitis Optica Spectrum Disorder Patients in Various Clinical Settings: A Single-Center Case Series with Concomitant Use of Rituximab

**DOI:** 10.7759/cureus.84703

**Published:** 2025-05-23

**Authors:** Ryota Amano

**Affiliations:** 1 Neurology, Fujiyoshida Municipal Hospital, Fujiyoshida, JPN

**Keywords:** ch50, neuromyelitis optica spectrum disorder (nmosd), ravulizumab, rituximab, satralizumab

## Abstract

Neuromyelitis optica spectrum disorder (NMOSD) is an autoimmune disease characterized by inflammation of the optic nerves and spinal cord, often associated with anti-aquaporin-4 antibodies. Ravulizumab (RVZ), a monoclonal antibody targeting complement protein C5, has shown promise in reducing relapse rates and preventing neurological deterioration. However, studies reporting its use in specific clinical circumstances, such as switching from or to other biologics, or during infectious episodes such as COVID-19, are scarce.

This case series presents four patients with NMOSD treated with RVZ, highlighting its effectiveness and safety in diverse scenarios. Notably, two patients transitioned from satralizumab to RVZ without relapse or elevated infection risk, supporting RVZ as a viable alternative. However, persistent neurological symptoms affecting quality of life, despite relapse control, emphasize the need for more comprehensive care beyond relapse prevention. All four patients received concomitant RVZ and rituximab (RTX) for a duration of six months, during which no notable adverse events were recorded. Furthermore, neurological symptoms that had affected their quality of life were alleviated by RTX. One patient with acute COVID-19 was successfully managed with RVZ, demonstrating its potential role during infectious episodes.

The study also questions the reliability of serum 50% hemolytic complement (CH50) levels for monitoring RVZ efficacy. Fluctuations in CH50 were observed, but all values normalized shortly after RVZ administration, suggesting that the timing of blood collection affects the results. CH50 levels above the detection threshold were observed only in samples collected immediately before the next RVZ administration. Therefore, it was considered appropriate to use samples obtained shortly after RVZ administration to assess the efficacy of RVZ based on CH50 levels.

In conclusion, RVZ offers effective relapse prevention and versatility in NMOSD management, even in challenging clinical contexts. In some cases, administration of RVZ resulted in mild improvements in symptoms such as pain, stiffness, and numbness. The transition from RVZ to RTX was achieved safely without the use of corticosteroids, as no increase in adverse events such as infections, nor any rise in NMOSD relapses during the initial phase of RTX induction, was observed, even during six months of concomitant administration. Further studies are warranted to evaluate whether the relapse rate in patients who switched to RTX using this method is comparable to that in patients who initiated RTX in combination with conventional steroids. Additionally, research is needed to establish safe switching strategies from RVZ to other biologic agents such as satralizumab or inebilizumab.

## Introduction

Neuromyelitis optica spectrum disorder (NMOSD) is an autoimmune disorder characterized by inflammation of the optic nerves and spinal cord, which often leads to severe neurological symptoms, including vision loss and paralysis. NMOSD is commonly associated with antibodies against aquaporin-4 (AQP4), a water channel protein expressed in the central nervous system. This disorder can have a relapsing course, necessitating timely and proper treatment to prevent long-term disability.

Recently, biological agents represent a significant advancement in the treatment of NMOSD, offering targeted therapy options that can improve patient outcomes. Ongoing research continues to explore the efficacy and safety of these treatments, particularly in different patient populations and under varying clinical circumstances [1].

Ravulizumab (RVZ) is a monoclonal antibody that inhibits complement protein C5, preventing its cleavage into C5a and C5b. RVZ, in contrast to its predecessor eculizumab (ECZ), is internalized via endocytosis and releases C5 under mildly acidic conditions (approximately pH 6.0), thereby facilitating lysosomal degradation of C5. ECZ exhibits low affinity for the neonatal Fc receptor (FcRn) and is consequently degraded within lysosomes. In contrast, RVZ binds to FcRn, enabling its recycling back into the circulation and thereby conferring an extended half-life relative to ECZ [2]. This mechanism is significant in conditions where complement activation plays a role in the pathophysiology, including NMOSD. In a phase 3 trial, RVZ showed a reduction in annualized relapse rates and prevented neurological deterioration compared with the placebo [3]. Furthermore, meta-analyses of clinical trial data have shown no significant differences in efficacy between ECZ and RVZ. RVZ is thus considered a therapeutic agent that maintains efficacy against NMOSD while allowing for an extended dosing interval compared to ECZ [4].

RVZ is a highly effective drug for preventing relapses in NMOSD; however, since it has been released recently, only a few studies have reported its use in special circumstances, such as switching from or to other biologics, or during the acute phase of coronavirus disease (COVID-19).

While it has been well established that measurement of the classical 50% hemolytic complement test (CH50) during ECZ administration rarely yields values above the detection threshold, thereby serving as a reliable marker for assessing pharmacological efficacy [5], the utility of serum CH50 monitoring in evaluating the therapeutic effectiveness of RVZ remains uncertain [6,7]. Moreover, some patients reportedly show detectable CH50 levels with suboptimal terminal complement inhibition of free C5 levels despite treatment with RVZ [7]. Furthermore, in the phase 3 trial, several patients were initially judged by their attending physicians to have experienced a relapse. Still, they were later determined by the independent review committee not to meet the criteria for relapse. Additionally, some patients showed temporary worsening of the Hauser Ambulation Index or the Expanded Disability Status Scale of Kurtzke [3]. Although these cases were not classified as clear relapses, it cannot be ruled out that minor astrocyte damage occurred due to mildly increased complement activity. Therefore, fluctuations in CH50 during RVZ administration may be associated with clinical worsening that does not meet the criteria for a definitive relapse. 

This report presents four cases demonstrating the use of RVZ under various real-world clinical circumstances. It mainly focuses on its efficacy, safety, and limitations, as well as observed fluctuations in CH50 levels under specific conditions.

## Case presentation

The patients’ representative profiles are shown in Table 1. For Patients 1 and 4, the effect of RVZ administration on clinical symptoms was unclear. Patients 2 and 3 showed mild improvement in clinical symptoms, but the improvement was not sufficient. All four patients transitioned from RVZ to RTX and underwent genetic studies. No mutations were identified in the c.2654G→A genetic variant, which is known to influence the binding affinity of ECZ and RVZ to complement component C5 [8].

**Table 1:** The patients’ representative profiles PSL; prednisolone, RTX; rituximab, RVZ; ravulizumab, STZ; satralizumab

Patient	Age/sex	Dose of RVZ (initial/maintenance)	Dose of PSL (before/after RVZ)	Biological agents before RVZ	Weeks of RVZ injection	Effect of RVZ on clinical symptoms	Effect of RTX on clinical symptoms
1	47/M	2700mg/3300mg	20mg/0mg	None	42	Not clear	Improvement in gait disturbance and sensory impairment
2	50/F	2400mg/3000mg	0mg/0mg	STZ	42	Mild improvement in bilateral thigh pain	Remission of bilateral thigh pain and hand stiffness
3	63/F	2400mg/3000mg	0mg/0mg	STZ	34	Mild improvement of numbness and stiffness in bilateral hands	Slight improvement in gait disturbance
4	64/M	2400mg/3000mg	0mg/0mg	None	42	Not clear	Slight improvement in numbness

**Figure 1:**
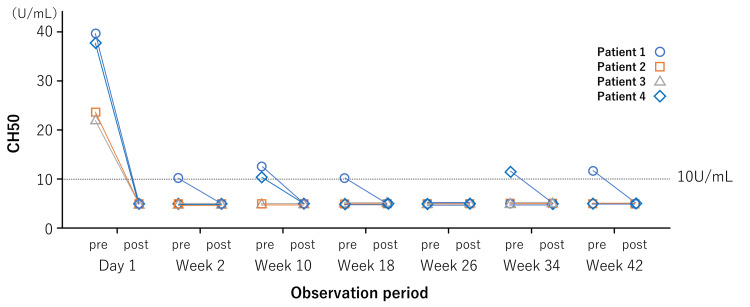
Timeline of CH50 in four patients Serum CH50 levels were decreased to below detectable levels (<10 U/mL) in all four patients on day 1. Patient 1 showed detectable CH50 levels in weeks 2, 10, 18, and 42. Moreover, Patient 4 showed detectable CH50 levels in weeks 10 and 34. However, no patients showed detectable CH50 levels after the administration of RVZ. Patient 2 and 3, viz. the cases of switching from satralizumab, did not show detectable levels of CH50 except for Day 1 (before the administration of RVZ). CH50, 50% hemolytic complement; RVZ, ravulizumab

To monitor CH50 (reference range: 30.0-46.0 U/mL), four adult patients with anti-AQP4-positive NMOSD treated with RVZ underwent evaluation of the serum CH50 levels before and after injection of RVZ on the same day. Blood collection was performed one hour after the completion of RVZ administration. Serum CH50 was assessed using the modified Mayer’s method (autoCH50-L Seiken II, Denka Seiken) and an automated analyzer (JCA-BM6050, Nihon Denshi). The results are shown in Figure 1. In summary, CH50 was occasionally detectable in two patients immediately prior to RVZ administration. However, CH50 levels fell below the detection limit one hour after RVZ administration in all patients. These fluctuations were considered to be influenced by the timing of blood collection.

Patient 1

This patient was previously reported in a single case report [9]. Briefly, a 47-year-old man with a prior diagnosis of anti-AQP4 antibody-positive NMOSD and a history of myelitis in his 30s presented to our hospital following his second relapse when he was 38 years old. After his seventh relapse, he was treated with oral prednisolone (PSL) at 10 mg/day and azathioprine (AZT) at 100 mg/day. In May 2023, he experienced left optic neuritis, which was his eighth relapse, and received high-dose methylprednisolone (1,000 mg/day for three days), which elicited substantial recovery from visual impairment.

**Figure 2:**
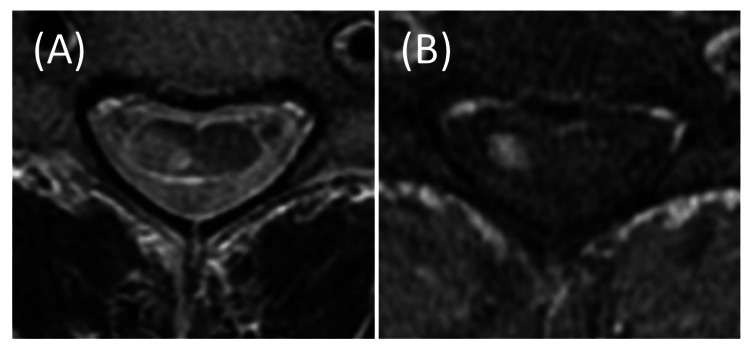
Cervical cord contrast-enhanced MRI in Patient 1 Axial T2-weighted (A) and T1 contrast-enhanced (B) images on admission. On T2-weighted image (A), a high signal intensity area was observed on the right side of the cervical spinal cord, with corresponding gadolinium enhancement in the same region (B). These findings are indicative of acute myelitis.

Due to his reluctance to increase the PSL dosage, the AZT dose was raised to 150 mg/day. Although treatment options involving antibody therapies were discussed to prevent further relapse, the patient was admitted to our hospital with gait disturbance and right hemiplegia, which had begun the day before admission. Cervical cord contrast-enhanced magnetic resonance imaging (MRI) revealed an acute-phase cervical cord lesion, confirming the ninth NMOSD relapse (Figure 2).

The patient was treated with high-dose methylprednisolone (1,000 mg/day for three days) followed by three sessions of plasma exchange (PE). After the third PE session, his symptoms improved, and he was discharged with a prescription of 20 mg/day of prednisolone. At that time, the top priority was to reliably prevent further relapses. Therefore, RVZ was chosen as the initial treatment. Although RVZ was initiated to prevent further relapse, it failed to yield significant neurological improvement. In an effort to alleviate residual neurological symptoms following relapse, RTX was initiated six months after the introduction of RVZ. This resulted in a modest improvement in both gait disturbance and sensory impairment over 2-3 months. RVZ was co-administered during the six months when recurrence is more likely with RTX treatment [10]. Over the 12-month period of RVZ treatment, including six months of concomitant administration with RTX, no relapses or adverse events were observed. Additionally, the dose of PSL was successfully tapered off within 12 weeks following the initial RVZ injection. Furthermore, no evident relapses or adverse events occurred during the six months after the transition to RTX monotherapy. In this case, the meningococcal vaccine was administered on two occasions: the first dose was given the day after completion of methylprednisolone pulse therapy, and the second dose was administered three weeks after the second administration of RVZ, as there were concerns that methylprednisolone pulse therapy and PE might diminish the efficacy of the first vaccination.

Patient 2

A 50-year-old woman, initially diagnosed with recurrent myelitis in 2007, experienced three relapses, two of which occurred in a cluster period between 2007 and 2008. After the relapse, anti-AQP4 antibodies were detected, and her diagnosis was revised to NMOSD. From 2007 to 2012, her relapses were managed with oral PSL. However, when her PSL dose was reduced to 10 mg/day in 2012, a relapse occurred, requiring her to remain on 15 mg/day until May 2021. Following the introduction of satralizumab (STZ) in 2021, PSL tapering was initiated.

By August 2023, while continuing STZ, her PSL dose was reduced to 3 mg/day. Around this time, the patient reported hand stiffness and thigh pain. Although the cause was unclear, her creatine kinase (CK) levels (reference range: 41-153 IU/L) rose from 120 IU/L to approximately 160 IU/L. Investigations for myositis-related autoantibodies and connective tissue diseases revealed no significant abnormalities. Despite her symptoms, the patient chose to discontinue PSL entirely, completing the taper by November 2023, retaining STZ as monotherapy.

Although she did not experience NMOSD relapses with STZ monotherapy, her hand stiffness and pain persisted, suggesting that while STZ was effective in preventing relapses, it was insufficient for improving her quality of life. In January 2024, her treatment was switched to RVZ, which was administered on the next scheduled STZ dose date. The meningococcal vaccine was administered one week after the final dose of STZ, which was three weeks before the first administration of RVZ.

Following the switch, her CK levels did not show a clear change, and hand stiffness remained unchanged; however, the thigh pain slightly improved, with reductions of 1.5 points on the Visual Analog Scale, 4 points on the Numerical Rating Scale, and 1 point on the Functional Rating Scale. This provided some relief while performing daily activities, although complete symptom resolution was not achieved. Six months after initiating RVZ, RTX was subsequently added, resulting in remission of both pain and stiffness within three months. At 16 months post-switch to RVZ, the patient remains relapse-free and has not experienced any adverse events. CK levels decreased to approximately 120 IU/L six months after starting RTX. 

For this patient, RVZ and RTX were co-administered for six months, akin to Patient 1. No relapses or adverse events have been observed in the four months following the transition to RTX monotherapy.

Patient 3

A 63-year-old woman with a history of left femoral head necrosis in 2013 initially presented with myelitis in 2001, leading to an initial diagnosis of multiple sclerosis. Between 2001 and 2016, she experienced two additional relapses of myelitis and one episode of optic neuritis. During this time, she was treated with interferon-beta (IFN-β) to prevent relapse. However, in 2016, anti-AQP4 antibodies were detected, and her diagnosis was revised to NMOSD. Since her condition was stable with IFN-β, the treatment was continued.

A third myelitis relapse in 2017 prompted the initiation of oral PSL. Although no further relapses occurred, she remained on PSL at 8 mg/day until May 2022. During a follow-up visit in June 2022, she reported moon facies, and STZ was introduced to enable PSL tapering. Nine months after starting STZ, PSL tapering was started, and the PSL dosage was gradually reduced to 2.5 mg/day by month 14. However, in the 16th month, the patient developed numbness and stiffness in both hands, along with pain in the right thigh, without evidence of relapse on MRI. The PSL dose was temporarily increased to 5 mg/day for symptom management.

At 18 months, she developed cellulitis with abscess formation in the left leg, requiring inpatient treatment. During hospitalization, necrosis of the right femoral head was identified, likely due to prolonged steroid use. Gradual PSL tapering was initiated, leading to successful discontinuation by month 19. At 24 months, she underwent right femoral head replacement, which alleviated the thigh pain, although the hand symptoms persisted.

To address the residual symptoms, she was transitioned from STZ to RVZ, which was administered on the next scheduled STZ dose date. The meningococcal vaccine was administered one week after the final dose of STZ, which was three weeks before the first administration of RVZ. Following RVZ treatment, she experienced mild improvement in the hand symptoms without NMOSD relapses or new infections during three months of follow-up; however, complete symptom resolution was not achieved.

In an effort to alleviate residual neurological symptoms, RTX was initiated three months after the introduction of RVZ. Although no marked improvement was observed in the symptoms affecting the hands, a slight improvement was noted in the gait disturbance that had originally been present as a sequela of prior myelitis after initiating RTX. No evident relapses or adverse events were observed during the six-month period of combined RTX and RVZ therapy.

Patient 4

**Figure 3:**
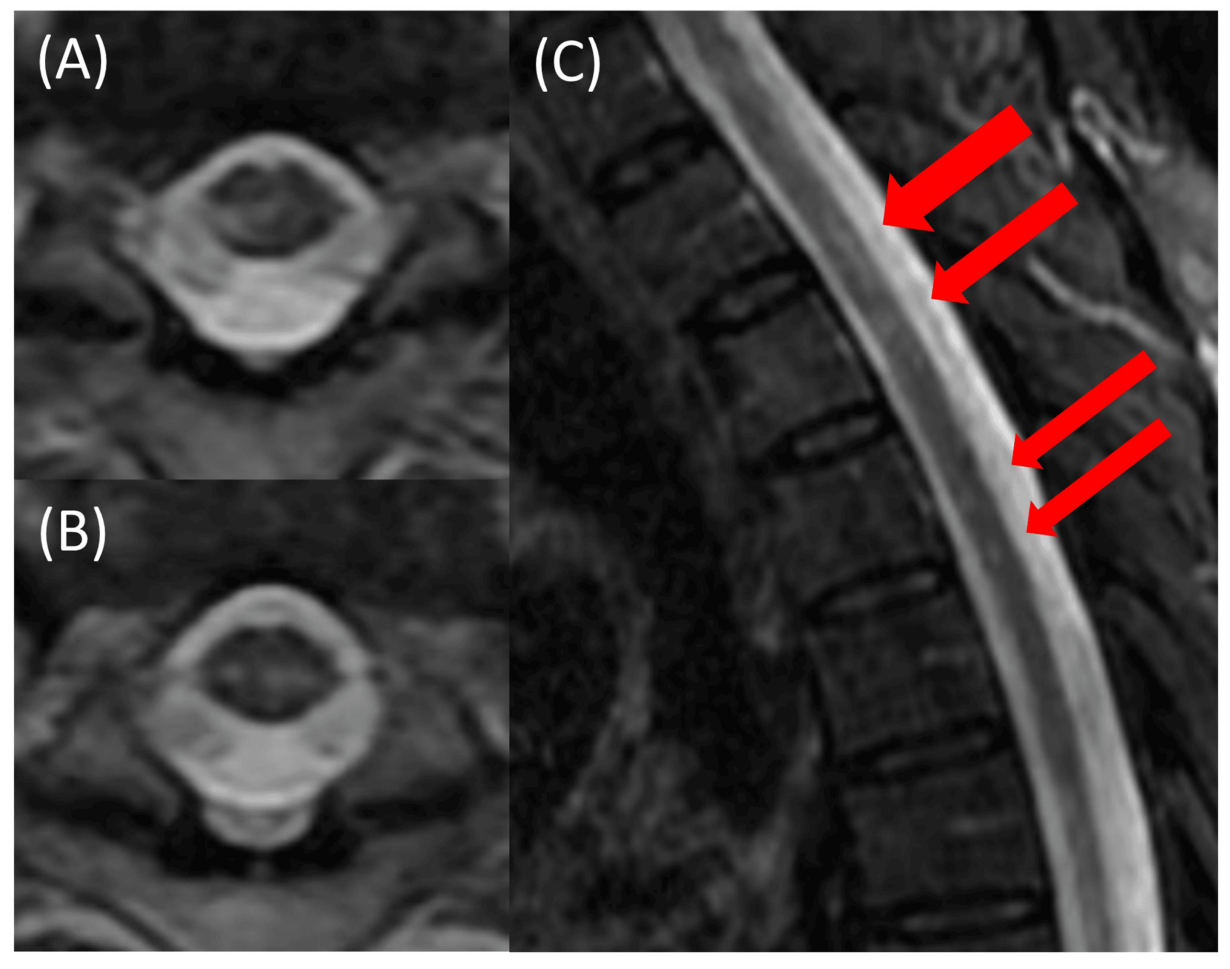
Thoracic spinal cord MRI in Patient 4 Thoracic spinal MRI showed a longitudinally extensive T2 hyperintense area with some discontinuities (A-C). The red arrows indicate the T2 hyperintense region. (A) and (B) correspond to the uppermost and lowermost slices at the locations of the arrows, respectively.

A 64-year-old man with a history of benign prostatic hyperplasia of unknown onset sustained a right femoral neck fracture in December 2023 after falling from scaffolding at work. He underwent screw fixation surgery. During his hospital stay in January 2024, he developed urinary retention and hiccups. In March, he reported numbness extending from the left back to the lateral chest, but no further evaluation was conducted, and he was discharged later that month. In April, the patient began experiencing painful tonic spasms. In early May, spinal MRI revealed myelitis (Figure 3), prompting a neurology consultation.

The patient was diagnosed with anti-AQP4 antibody-positive NMOSD and admitted in late May. At admission, urinary retention had resolved, but numbness in the left back and lateral chest persisted, along with severe impairment of vibration and proprioception below the Th8 level. Although he could walk, tandem gait was not possible. High-dose methylprednisolone (1,000 mg/day for three days) was administered, improving his ability to perform tandem gait. Five days after completing steroid therapy, a fellow patient tested positive for SARS-CoV-2; our patient developed mild throat pain and was subsequently tested and confirmed positive for COVID-19. Due to the subacute phase of NMOSD, difficulties in conducting PE during isolation, and concerns about initiating STZ or B-cell depletion therapies during the acute phase of COVID-19, RVZ was started for relapse prevention. The meningococcal vaccine was administered 13 days after the initial dose of RVZ, on the day before the second dose, at a time when the patient's COVID infection was considered to have resolved. Oral PSL was not used. RVZ did not lead to significant neurological improvement. One month after the initiation of RVZ, the patient developed acute cystitis caused by *Staphylococcus aureus*, which was successfully treated with intravenous ceftriaxone and oral levofloxacin. Three months after starting RVZ, he underwent femoral head replacement surgery for avascular necrosis, with no perioperative complications. To alleviate residual neurological symptoms, specifically, numbness extending from the left back to the lateral chest, RTX was initiated five months after the introduction of RVZ. A slight improvement in the numbness was observed two to three months after RTX initiation. In the six months following the commencement of combined RVZ and RTX therapy, no NMOSD relapses or other adverse events were reported.

## Discussion

This case series highlights the complexities of managing anti-AQP4 antibody-positive NMOSD and experiences of using RVZ under various clinical circumstances. Moreover, I have reported the clinical changes observed in patients following the switch to either RVZ or RTX. Below, I discuss each of these observations.

Safety and effectiveness of RVZ in various clinical situations

Transition From STZ

One key insight gleaned from these cases is the effectiveness and safety of RVZ in diverse clinical situations, including patients transitioning from STZ. Patients 2 and 3, who switched from STZ to RVZ, did not experience NMOSD relapses and infections during the follow-up period, suggesting that changing from STZ to RVZ is a viable option when needed. In these cases, relapse prevention was well-managed with STZ monotherapy; however, they were struggling with residual symptoms of NMOSD. While switching directly from STZ to inebilizumab (INB) or RTX was considered, STZ is a recycling antibody and remains in the body for a certain period even after discontinuation. This raised significant concerns about an increased risk of infection due to excessive immunosuppression when combined with B-cell targeted therapies, as well as the possibility that relapse of NMOSD might be triggered once the effect of STZ wears off. On the other hand, a transition to RVZ was expected to carry a lower risk of infection and relapse, making it a safer option for switching treatment. Therefore, I first transitioned to RVZ, before B cell depletion therapy. However, these cases also indicate that RVZ alone may not fully cover all aspects of patient care. For instance, both patients reported persistent symptoms such as numbness, stiffness, or thigh pain despite the absence of relapses, which underscores the limitations of relapse-focused therapy in improving the quality of life and functional outcomes.

RVZ Use in the Acute Phase of COVID-19

The case of Patient 4 highlights the unique challenges of managing NMOSD during the acute phase of COVID-19. In this situation, RVZ was chosen over other immunotherapies due to the difficulty in initiating STZ or B-cell depletion therapies during active infection and the logistical challenges of conducting PE in isolation. RVZ has been tested as a treatment for COVID-19, and its safety has already been confirmed [11]. This case emphasizes the potential role of RVZ as a safer option during infectious episodes, though further studies are needed to validate its use under similar conditions.

Short-term combination with RTX

In all patients, RTX could be co-administered with RVZ. One study has reported short-term combined use of eculizumab and RTX in sickle cell disease [12]. There have been no prior reports of combining or transitioning between complement inhibition therapies such as ECZ or RVZ and INB, even for a short period. Given the potential risk of NMOSD relapse within 6 months following such a transition, and after thorough discussion with the patients, we decided to proceed with a switch from RVZ to RTX, incorporating a brief overlap period to reduce that risk. While the long-term safety of RVZ and RTX remains uncertain, short-term use, until RTX fully exerts its therapeutic effect, may be relatively safe. Moreover, in this study, the number of peripheral blood CD19-positive cells was assessed in all cases six months after RTX administration, which was confirmed to be 0 cells/µL. At least during the first six months following induction, RTX maintained its efficacy, and no increase in infectious complications was observed even when used in combination with RVZ.

The association of C5 inhibition and pain 

C5 inhibitors, including RVZ, can alleviate pain by targeting C5a, a key component of the complement system involved in immune responses and inflammation. C5a, a pro-inflammatory molecule, binds to C5aR expressed on immune and non-immune cells, leading to the release of pain-mediating cytokines and recruitment of inflammatory cells such as neutrophils. Inhibiting C5a/C5aR signaling reduces neuroinflammation, peripheral sensitization, and glial cell activation, which are linked to inflammatory, postoperative, and neuropathic pain. Therefore, modulation of this pathway offers potential therapeutic options for controlling chronic pain states [13].

HyperCKemia amelioration by RTX in Patient 2

It is difficult to fully explain why Patient 2 experienced a decrease in CK levels after switching to RTX. However, pathological studies confirmed that hyperCKemia associated with NMOSD was not due to muscle fiber necrosis. Two mechanisms have been proposed to explain this phenomenon:

The first mechanism involves complement-mediated damage to the sarcolemma by AQP4-IgG, leading to the leakage of free CK from the cytoplasm into the bloodstream. Cytoplasmic CK is largely unbound to the cytoskeleton. Meanwhile, AQP4 is anchored in the sarcolemma as part of the dystrophin-associated protein complex that links the cytoskeleton to the extracellular matrix. Disruption or structural disorganization of the sarcolemma due to complement activation could result in CK leakage into the serum [14].

The second mechanism suggests that AQP4 dysfunction negatively affects energy metabolism pathways and intracellular calcium regulation in muscle cells. In AQP4-knockout mice, altered expression of proteins involved in energy metabolism and calcium handling has been observed in muscle tissue, indicating a physiological role for AQP4 in these processes [15]. When AQP4 is functionally impaired by AQP4-IgG, it may lead to metabolic and structural damage in the muscle, resulting in CK leakage into the bloodstream [14].

In the case of Patient 2, the first mechanism alone cannot explain the rise in CK levels, particularly considering that RVZ strongly suppresses immune complex activation. Therefore, the second mechanism is presumed to have played a predominant role. It has also been reported that AQP4-IgG can induce internalization of AQP4 through a non-complement-dependent pathway, suggesting that these antibodies do not exclusively activate immune complexes [16].

It is possible that the second mechanism contributed to the observed CK elevation. Subsequently, RTX administration suppressed the production of AQP4 antibodies, leading to normalization of CK levels. This hypothesis may also explain why Patient 2 did not experience hyperCKemia while taking more than 3 mg/day of PSL.

However, this remains a speculative explanation, and further studies involving multiple NMOSD patients presenting with hyperCKemia are needed. Additionally, mild pain improvement after RVZ administration, despite unchanged CK levels, may be attributable to an analgesic effect related to C5a [13].

The alleviation of numbness, stiffness, and pain by RTX

The mechanism by which RTX alleviates numbness, stiffness, and pain remains unclear. However, INB, another B-cell depletion therapy, has been suggested to potentially improve quality of life, especially in pain, in patients with NMOSD [17]. Additionally, both INB and RTX have been reported to improve residual visual impairment [18], suggesting that RTX, like INB, may also have the potential to improve quality of life. In an in vitro experimental system, treatment of human iPSC-derived astrocytes with AQP4 antibodies induces structural alterations in mitochondria and their associations with the endoplasmic reticulum and lysosomes at the ultrastructural level in a complement-independent manner, which potentially leads to impaired mitochondrial functions and dynamics [16]. Unlike RVZ, which suppresses complement activation, RTX suppresses the production of AQP4 antibodies. Assuming that this suppression contributed to the prevention of complement-independent astrocyte dysfunction, it is conceivable that such an effect may have played a role in ameliorating residual symptoms of NMOSD, including numbness, stiffness, and pain. However, the improvement of residual symptoms cannot be expected in all patients treated with RTX. Therefore, when introducing RTX with the aim of addressing residual symptoms, further investigation is warranted to identify the patient populations for whom this approach is most appropriate.

CH50 and RVZ

The variability in serum CH50 levels observed among the patients raises questions about the utility of CH50 as a biomarker for monitoring RVZ efficacy. Although the data are derived from studies in PNH, it has been reported that in ECZ-treated patients, CH50 activity is directly associated with circulating free ECZ levels and negatively correlated with elevated levels of LDH and bilirubin, which are indicators of red blood cell destruction [19]. However, in the case of RVZ, in contrast to ECZ, some patients are reported to show detectable CH50 levels during treatment [7], whereas free C5 levels stably decreased to below complete terminal complement inhibition. Therefore, in the CHAMPION-NMOSD study, the pharmacological effect was assessed via serum-free C5 concentrations [3]. However, Cataland et al.’s study mentions various CH50 assays, and the threshold for assessing significant complement blockage varies in each assay [7]. Moreover, because the concentrations of serum RVZ are the highest just after administration and the lowest just before the next administration, the timing of blood collection is considered to affect the results of CH50. In this study, CH50 levels were detectable only before the administration of RVZ, and decreased to below detectable levels after administration. Although it is possible that RVZ efficacy may slightly diminish right before the next scheduled dose in certain cases, the CH50 values only slightly exceeded 10 U/mL, raising questions about how (or if) these fluctuations are associated with NMOSD relapse. 

In the two patients (Patient 1 and Patient 4) whose CH50 levels were measured above the detection threshold, no clear association was observed between the elevated CH50 and clinical symptoms, and neither patient reported any worsening of neurological symptoms immediately prior to the subsequent RVZ administration. Unlike Patients 2 and 3, these two patients did not exhibit any noticeable improvement in neurological symptoms such as numbness or pain; however, this may have been due to the initiation of RVZ during the subacute phase following a relapse, making it difficult to distinguish between recovery from the relapse and any new clinical changes. In any case, the occasional measurement of CH50 above the detection threshold in patients receiving RVZ may be related to subclinical disease activity or symptom worsening that does not meet the criteria for definite relapse, as observed in the phase 3 trial [3]. Further studies are warranted to investigate this possibility.

To ensure that RVZ efficacy is adequately assessed using the modified Mayer’s method, measuring CH50 levels approximately 1 hour after RVZ administration may be optimal. This approach could provide more consistent results and confirm whether terminal complement inhibition has been effectively achieved.

Limitations

However, this study has several limitations. First, no patients received combination therapy with RVZ and RTX for more than six months, making it difficult to assess the safety of such long-term use. Second, the observation period following the switch to RTX monotherapy was relatively short, so the long-term efficacy in preventing relapses remains uncertain. This latter point is particularly important, as none of the patients who transitioned to RTX in this study were administered PSL concurrently at the time of RTX initiation. Therefore, it is essential to examine whether relapse rates differ in patients who start RTX therapy with PSL, as is typically done in clinical practice.

Furthermore, regarding the improvement in quality of life observed with RVZ and RTX, most of the evaluations relied on patients’ subjective impressions, and objective assessments using appropriate measures were not conducted. In addition, no placebo-controlled group was included, making it difficult to determine whether the observed effects can be attributed to the pharmacological action of the drugs themselves.

## Conclusions

In conclusion, RVZ is effective for preventing NMOSD relapse, even in patients transitioning from other biologics. Furthermore, even with a switch from STZ or combination with RTX, there was no clear increase in infections, at least in the short term, and total hip replacement was also successfully performed without any recurrence. RVZ is considered a highly versatile drug, not only for its potent effect in preventing NMOSD relapse but also for its adaptability to various clinical situations. 

Further exploration of the practical use of RVZ in clinical settings is warranted in the future. This includes evaluating its efficacy when administered during the initial onset or acute relapse phase of the disease and establishing safe and effective strategies for transitioning to STZ or INB without triggering relapse.
